# GP73-regulated oncolytic adenoviruses possess potent killing effect on human liver cancer stem-like cells

**DOI:** 10.18632/oncotarget.8830

**Published:** 2016-04-18

**Authors:** Xinmin Zhang, Shulin Meng, Rong Zhang, Buyun Ma, Tao Liu, Yu Yang, Wenjie Xie, Xianglei Liu, Fang Huang, Tao Liu, Xiumei Zhou, Xinyuan Liu, Yigang Wang

**Affiliations:** ^1^ Xinyuan Institute of Medicine and Biotechnology, School of Life Sciences, Zhejiang Sci-Tech University, Hangzhou 310018, PR China; ^2^ Institute of Biochemistry and Cell Biology, Shanghai Institutes for Biological Sciences, Chinese Academy of Sciences, Shanghai 200031, PR China; ^3^ Central China Normal University, Wuhan 430079, China; ^4^ School of Public Health, Zhejiang University, Hangzhou 310058, China; ^5^ Department of Otolaryngology, Zhujiang Hospital, Southern Medical University, Guangzhou 510282, China; ^6^ Department of Gastroenterology and Hepatology, Erasmus MC-University Medical Center, Rotterdam 3015, Netherlands

**Keywords:** GP73, oncolytic adenovirus, liver cancer stem-like cells

## Abstract

Cancer stem cells (CSCs), also known as tumor-initiating cells, are highly metastatic, chemo-resistant and tumorigenic, and are critical for cancer development, maintenance and recurrence. Oncolytic adenovirus could targetedly kill CSCs and has been acted as a promising anticancer agent. Currently, a novel GP73-regulated oncolytic adenovirus GD55 was constructed to specifically treat liver cancer and exhibited obvious cytotoxicity effect. However, there remains to be confirmed that whether GD55 could effectively eliminate liver CSCs. We first utilized the suspension culture to enrich the liver CSCs-like cells, which acquires the properties of liver CSCs in self-renewal, differentiation, quiescence, chemo-resistance and tumorigenicity. The results indicated that GD55 elicited more significant cytotoxicity and stronger oncolytic effect in liver CSC-like cells compared to common oncolytic virus ZD55. Additionally, GD55 possessed the greater efficacy in suppressing the growth of implanted tumors derived from liver CSC-like cells than ZD55. Furthermore, GD55 induced remarkable apoptosis of liver CSC-like cells *in vitro* and *in vivo*, and inhibited the propogation of cells and angiogenesis in xenograft tumor tissues. Thus, GD55 may virtually represent an attractive therapeutic agent for targeting liver CSCs to achieve better clinical outcomes for HCC patients.

## INTRODUCTION

Cancer stem cell (CSC) model has provided a novel cellular mechanism that contributes to phenotypic and functional heterogeneity in diverse tumors [1, 2]. The hypothesis posits that CSCs reside at the apex of a cellular hierarchy and generate more differentiated non-CSC progeny. In these cases, CSCs are thought to be responsible for driving tumor growth, and disease recurrence through therapy resistance and metastasis [3]. Therefore, CSCs are key determinants in the process of metastatic dissemination and disease relapse of some cancer therapy, and crucial targets for the complete elimination of numerous cancers, including liver cancer [4].

Liver cancer is the second most deadly and fifth most common cancer in men worldwide [5]. Hepatocellular carcinoma (HCC) and Intrahepatic cholangiocarcinoma (ICC) are first and second most frequent type of liver cancer, respectively [5]. Both of which are found to be phenotypic and functional heterogeneous diseases, supporting the existence of liver CSCs [6]. Indeed, the concept of liver CSCs have been further validated in many aspects of features, including self-renewal capacity, tumorigenicity, chemotherapy and radiation resistance, responsible for liver cancer relapse after therapy [4]. Thus, these indicate that the need to specifically target the liver CSCs holds extremely importance for eradicating liver tumor.

In contrast to conventional treatments that can eliminate the bulk of cancer cells within a tumor while leaving behind a small subpopulation of CSCs that proceed to drive tumor relapse, oncolytic virotherapy as an emerging treatment approach are effective in destroying cancer cells in both preclinical models and clinical trials [7]. Oncolytic viruses can effectively kill infected cancer cells via multiple ways, including direct oncolysis of cancer cells and indirect killing of uninfected cancer cells such as destruction of tumor blood vessels and inducement of antitumor immunity [8, 9]. Moreover, oncolytic viruses targeting CSCs are not subject to the mechanisms of drug resistance for traditional treatments [10]. For example, adenovirus, an oncolytic virus, is able to effectively infect both highly proliferative cells (non-CSCs) and quiescent cells (CSCs), and not is pumped out of infected cells by the ATP-binding cassette (ABC) transporters compared to chemotherapy drugs [11–13].

In the last few years, oncolytic adenovirus represents a promising agent for treatment of many cancers. In our previous studies, Cancer Targeting Gene-Virotherapy (CTGVT) strategy displayed greater antitumor effect in cancer therapy compared with oncolytic virotherapy [14, 15]. ZD55, a previously modified oncolytic adenovirus system, was designed based on the CTGVT strategy [16]. It is able to selectively replicate in cancer cells and exert apparently cytotoxicity effects on them, and also can carry multiple types of exogenous antitumor genes to strengthen its cancer-killing effect [16]. Currently, based on ZD55, we constructed a novel GP73-regulated oncolytic adenovirus GD55, which acquired stronger cytotoxicity effect on liver cancer cells than ZD55, suggesting that GD55 might be a valuable agent in treating liver cancer [17]. GP73, a Golgi membrane glycoprotein, also called GOLPH2, was demonstrated as an excellent marker for HCC diagnosis in recent years, and even it showed the higher sensitivity and specificity than liver cancer marker α fetoprotein (AFP) [18]. Here, we proceeded to verified whether GD55 could also effectively destroy liver CSCs rather than do that only for non-CSCs reported previously by us [17]. Our present results indicated that GD55 could significantly elicit cytotoxicity in liver CSC-like cells enriched in suspension culture *in vitro* and *in vivo*, and exhibited more obvious killing-effect than that of ZD55.

## RESULTS

### Liver cancer cells through suspention culture acquire the properties of CSCs

**Figure 1:**
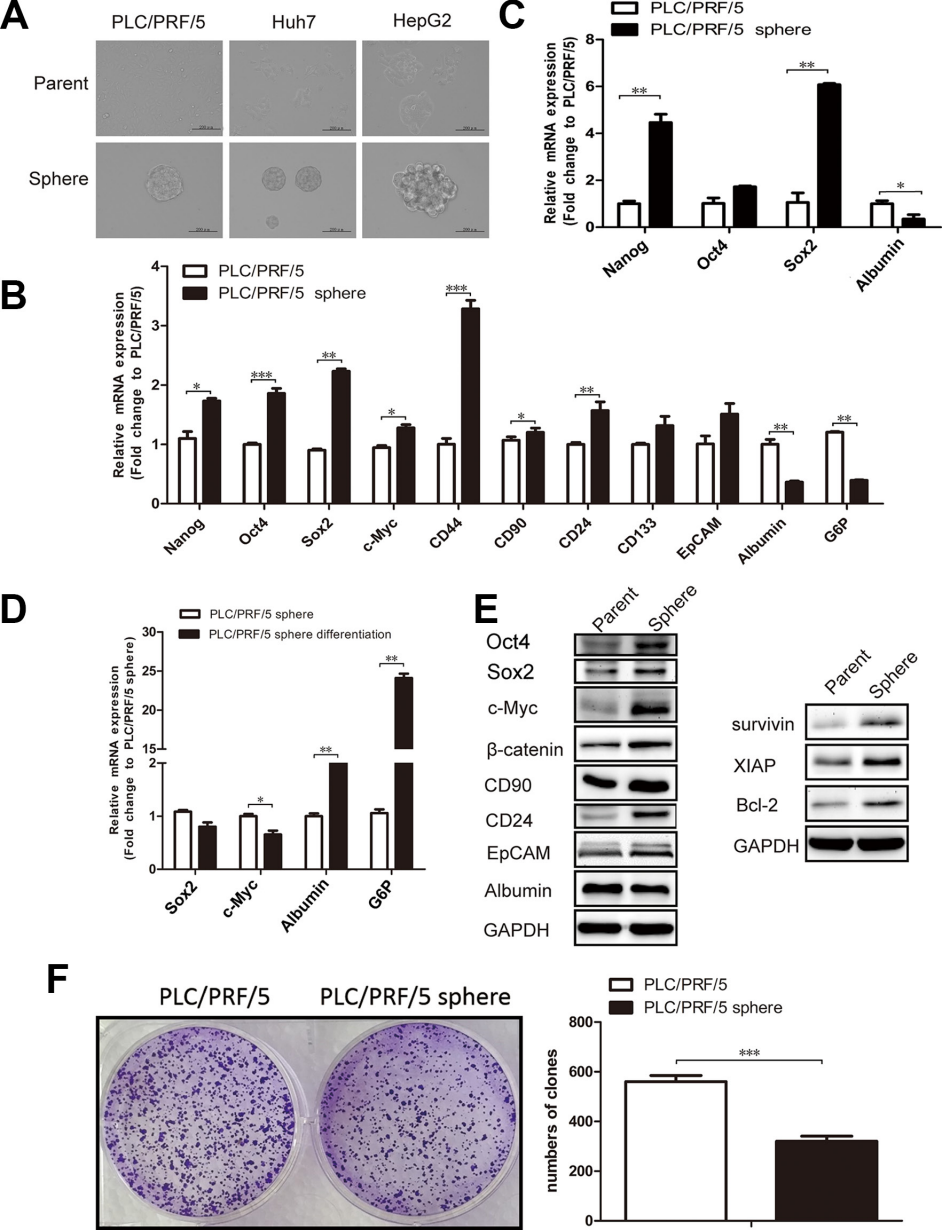
PLC/PRF/5 sphere cells possess multiple properties associated with liver CSCs (**A**) Hepatoma cell lines, including PLC/PRF/5, Huh7 and HepG2, could form sphere bodies in suspension culture with conditioned medium. Scale bar = 200 μm. (**B**) PLC/PRF/5 sphere cells overexpressed many liver CSCs-associated genes (e.g., Nanog, Oct4, Sox2) and downregulated mature hepatocyte markers (e.g., Albumin, G6P) when compared to PLC/PRF/5 cells. qRT-PCR data were normalized to GAPDH gene and are shown as fold change relative to PLC/PRF/5 cells. (**C**) PLC/PRF/5 sphere-forming cells of the 3rd generation sustained a high mRNAs expression level for Nanog, Sox2. (**D**) The mRNA levels for SOX2, c-Myc, Albumin and G6P were measured by qRT-PCR in PLC/PRF/5 sphere cells and their differentied counterparts after cultured 7 days in serum adherent condition. (**E**) Protein level of liver CSCs-associated genes, mature hepatocyte marker, and anti-apoptosis genes in PLC/PRF/5 parental or sphere cells. (**F**) PLC/PRF/5 sphere cells formed fewer colony numbers (> 70 cells/clone) by crystal violet staining. All experiments were repeated three times and all data shown represented mean ± SD (*n* = 3). *P < 0.05, **P < 0.01, ***P < 0.001.

The suspension culture in growth factor-defined serum-free medium can screen and enrich the cells associated with the traits of CSCs [12, 13, 19–21]. To valitate whether liver cancer cell lines could acquire these traits when passed through the suspension culture, three HCC cell lines PLC/PRF/5, Huh7 and HepG2 were subjected to specific serum-free medium as described in materials and methods. In ultra-low detachment plates, all these three cell lines gradually formed non-adherent spheroid bodies after culture for 4–6 days (Figure 1A), which are named as sphere cells relative to their parental cells cultivated adherently in a single layer. As anticipated, the expression of multiple liver CSCs-associated genes that related to self-renewal and surface antigen markers were increased in PLC/PRF/5 sphere cells compared to their parental cells (Figure 1B); while the levels of mRNAs encoding the mature hepatocyte markers (such as Albumin and G6P) were apparently decreased in PLC/PRF/5 sphere cells (Figure 1B). PLC/PRF/5 sphere cells expanded in suspension culture for 3 passages were capable of maintaining an even higher mRNAs expression for Nanog and Sox2 (Figure 1C). In contrast, PLC/PRF/5 sphere cells could gradually revert back to their parental counterparts after culturing adherently in serum medium in 7th day (Figure 1D, Supplementary Figure S1A), but not in 4th day (Supplementary Figure S1B). We further verified that some of the related proteins encoded by the above mentioned genes functioned in liver CSCs are upregulated in PLC/PRF/5 sphere cells and, simultaneously, that the expression of anti-apoptosis proteins for XIAP, Survivin and Bcl-2 is increased in PLC/PRF/5 sphere cells (Figure 1E). In principle, the elevated expression patterns for pleiotropic acting transcription factors (such as Nanog, Sox2 and OCT4) operated in CSCs should be closely echoed by corresponding activated signaling circuits in which the related signaling proteins were phosphorylated to transmit signals to induce the gene transcription. Indeed, sphere cells obtained the higher phosphorylation levels of STAT3 and AKT than their PLC/PRF/5 parental cells (Supplementary Figure S2A). To compare the relative quiescence of sphere and parental cells in PLC/PRF/5, cell proliferation rate was measured by Colony formation assay. The result showed that sphere cells proliferate at a significantly lower rate than parental cells (Figure 1F). The fluorescence-activated cell sorting (FACS) analysis data also showed that the ratio of quiescent G0/G1 phase cells increase in PLC/PRF/5 sphere cells (Supplementary Figure S3). We also found that the mRNAs encoding the epithelial-mesenchymal transition (EMT)-inducing markers are up-regulated in the sphere cells (Supplementary Figure S2B), indicating the enhanced cancer metastatic capacity.

**Figure 2:**
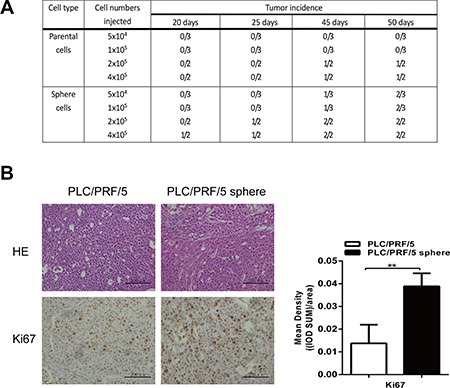
Sphere-forming cells exhibited higher tumorigenicity *in vivo* (**A**) Tumorigenicity experiments of PLC/PRF/5 sphere-forming cells and parental cells in NOD/SCID mice, PLC/PRF/5 sphere cells exhibited significantly stronger tumor-initiating ability. (**B**) Histologicaland IHC analysis of tumors from PLC/PRF/5 cells and PLC/PRF/5 sphere cells. Hematoxylin and eosin staining of a subcutaneous tumor (upper) and IHC staining of the tumor with anti-Ki-67 (lower). Scale bar = 200 μm. A column drawing represent statistic data for mean density of 5 fields of view. Data was analyzed using IPP 6.0 image analysis software.

Generally, CSCs are defined operationally by their robust ability to initiate new tumors *in vivo*. To evaluate the tumorigenicity in mice, various quantity of PLC/PRF/5 sphere and parental cells were subcutaneously implanted into the left or right rear of NOD/SCID mice, respectively. As anticipated, PLC/PRF/5 sphere cells possess significantly stronger tumor-initiating ability and higher proliferative rate than parental cells in mice (Figure 2A and 2B). These above results indicated that the sphere cells hold the attributes of liver CSCs.

### Sphere cells display an apparent resistance to conventional anti-tumor agents, but not to thioridazine and ZD55

**Figure 3:**
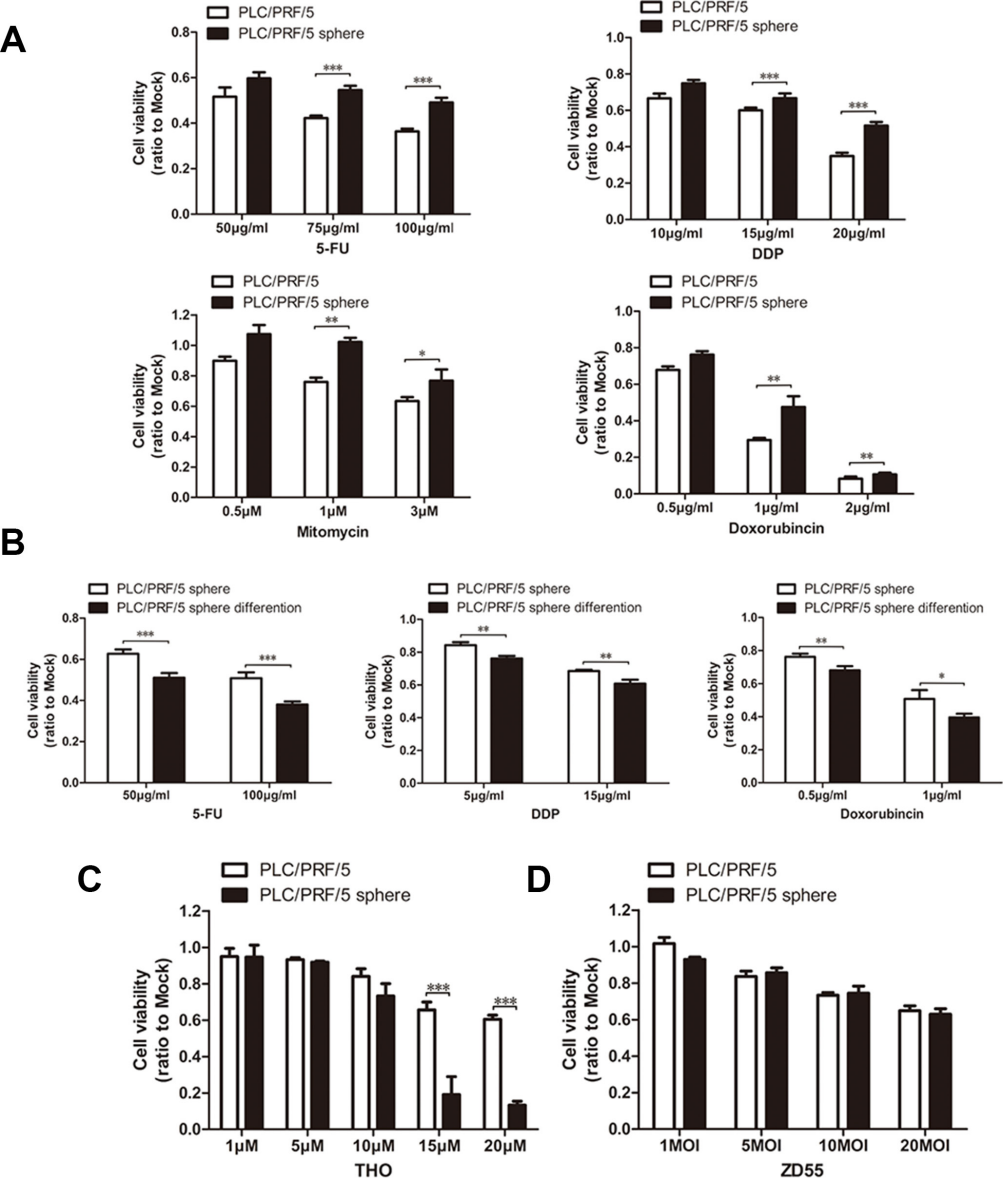
Sphere-forming cells were resistance to conventional chemotherapeutics *in vitro*, but not to THO and ZD55 (**A**) PLC/PRF/5 sphere cells displayed apparent resistance to cytotoxic chemotherapy than PLC/PRF/5 cells and (**B**) PLC/PRF/5 sphere-differentiated cells after treatment with 5-FU, DDP, doxorubincin and with or without mitomycin for 2 days. Cell viability was detected with MTT assay and repeated for three times. The relative cell viability was shown by fold change to the corresponding mock. The cytotoxicity effects of THO (**C**) and ZD55 (**D**) on both sphere cells and parental cells in PLC/PRF/5. Cell survival was determined by MTT assay. All experiments were repeated three times and all data shown represented mean ± SD (*n* = 3). *P < 0.05, **P < 0.01, ***P < 0.001.

CSCs exhibited significant resistance to traditional anti-tumor drugs. We used the cell stability assay to investigate whether the PLC/PRF/5 sphere cells acquired the property of chemo-resistance. The results showed that the PLC/PRF/5 sphere cells are significantly resistant to cytotoxic chemotherapy 5-FU, cis-platinum (DDP), doxorubincin and mitomycin compared to their parental cells or their sphere-differentiated counterparts (Figure 3A and 3B), and indeed, the related mRNA of drug-resistance genes were elevated in sphere cells (Supplementary Figure S2C). Thioridazine (THO), a recent intensely-studied drug for neoplastic cells, could specifically target and impair human blood CSCs through dopamine receptors which selectively express on the surface of CSCs [22]. In our experiments, THO displayed strong cytotoxicity to the sphere cells but weak to their parental cells (Figure 3C), and synchronously, we observed that the DR5 protein (the killer/death receptor 5, one of dopamine receptors) level increase in PLC/PRF/5 sphere cells (Supplementary Figure S2D), which inferred the killing effect of THO to CSCs. Nevertheless, unlike traditional anti-tumor drugs and THO, the common oncolytic virus ZD55 with the characteristic of cancer-killing virus, had a similar cytotoxicity effect on both sphere cells and parental cells in PLC/PRF/5 (Figure 3D), and no obvious behavior of the resistence was observed.

Our data further indicated that some of liver cell lines (such as PLC/PRF/5) acquired many properties of liver CSCs through suspension culture, that is sphere cells, which confer the sphere cells resistance to conventional anti-tumor agents, but sensitivity to THO and ZD55. Especially, in some ways it was plausible that ZD55 might represent the more superiority relative to the used anti-cancer agents.

### GP73-regulated oncolytic adenovirus exhibit enhanced cytotoxic effect on PLC/PRF/5 sphere cells

**Figure 4:**
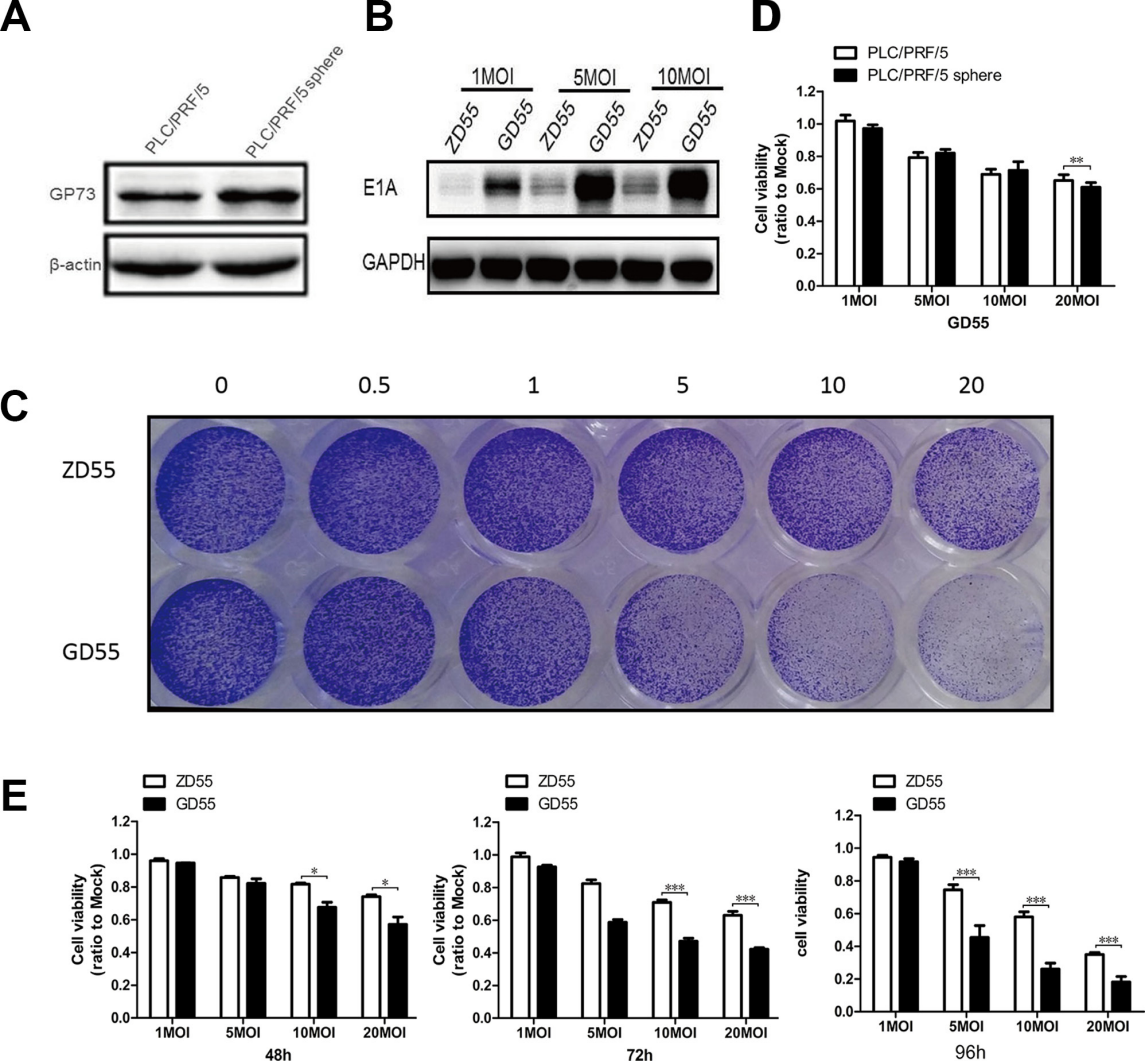
Analysis of infection efficiency and cytotoxicity of GP73-modified adenoviruses on PLC/PRF/5 sphere cells (**A**) GP73 expression was detected in PLC/PRF/5 cells and PLC/PRF/5 sphere cells (**B**) E1A expression was detected in PLC/PRF/5 sphere cells after treated with ZD55, GD55 for 2 days at 1, 5, 10 MOI. (**C**) GD55 showed enhanced cytotoxicity in PLC/PRF/5 sphere cells. PLC/PRF/5 sphere cells were treated with indicated MOI (0.5, 1, 5, 10, 20) of ZD55 and GD55 for 2 days, respectively, and subjected to crystal violet staining for cell viability determination. (**D**) GD55 held a similar killing efficacy PLC/PRF/5 sphere cells and their parental cells determined by MTT assay. (**E**) Comparison on cell viability of PLC/PRF/5 sphere cells treated with ZD55 and GD55 at indicated MOI for second, third, fourth day.

Previous reports indicated the high expression of GP73 in hepatocellular carcinoma cells [17–18]. Furthermore, we have demonstrated that GP73-regulated oncolytic adenovirus exerted potent antitumor efficacy in hepatocellular carcinoma [17]. Nevertheless, it is yet to be proven whether GD55 could effectively eliminate liver CSCs (such as the sphere cells) in the same way as we have reported for liver cancer cells, and our present results also showed that PLC/PRF/5 sphere cells acquired a little more expression level of GP73 (Figure 4A and Supplementary Figure S4A), might indicating potent killing effect on human liver cancer stem-like cells for GD55. The construction of the recombinant adenoviruses were depicted in Supplementary Figure S4B, which were packaged and amplified in HEK-293 cells in order to fulfill the related experiments.

To investigate the infection ability and cytotoxicity of GD55 and ZD55 on sphere cells, PLC/PRF/5 sphere cells were respectively treated with GD55 and ZD55 in indicated MOIs. Results showed that the more E1A (the first important early gene from adenovirus during replication) protein level and stronger cytotoxicity was observed in GD55-treated group compared to ZD55 (Figure 4B and 4C), implying the superiority of GD55. In addition, GD55 also exhibited a nearly equal killing efficacy to PLC/PRF/5 sphere cells and their parental cells as did ZD55 (Figure 4D). Furthermore, we used the MTT assay to test the survival rate of the sphere cells after being treated with GD55 and ZD55, respectively. The cytotoxicity effect of GD55 on PLC/PRF/5 sphere cells was much more obvious than that of ZD55 in indicated time points and various MOIs (Figure 4E, Supplementary Figure S4C and S4D). The results showed that GD55 presented increased inhibitory effect on liver CSCs proliferation, and exerted stronger cytotoxicity effect for PLC/PRF/5 sphere cells over the prolonged infection time.

**Figure 5:**
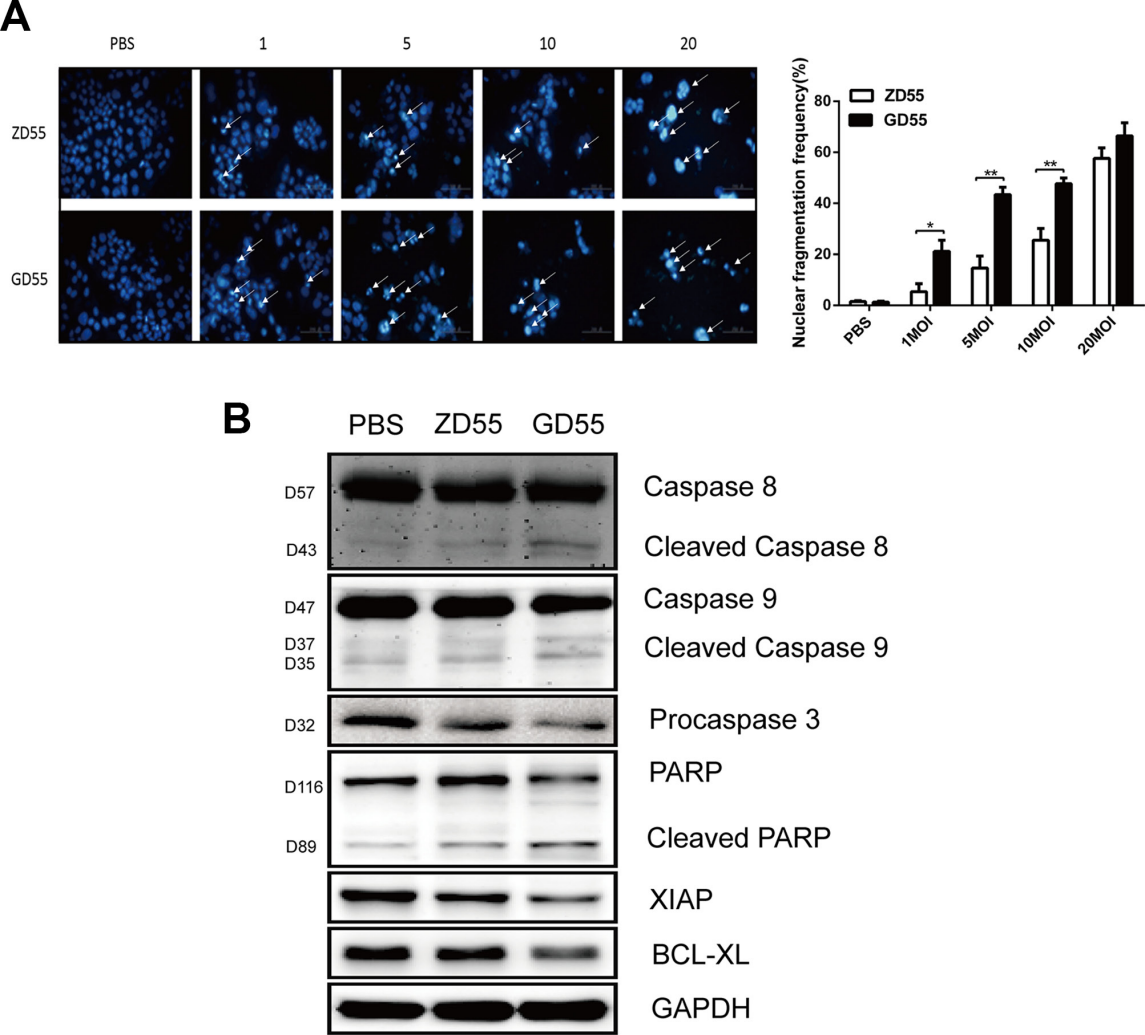
GD55 could induce the more extent of apoptosis in PLC/PRF/5 sphere cells compared to ZD55 (**A**) Increased nucleic fragmentation (arrow) was observed in PLC/PRF/5 sphere cells after 2 days treatment of ZD55 or GD55 at 1, 5, 10, 20 MOI as detected by hoechst staining. Scale bar = 200 μm. A column drawing representing statistic data for three repeats. The percentage (%) = Number of nucleic fragmented cells in six fields/Number of total cells in six fields. (**B**) Western blot analysis of the apoptosis-associated proteins. GAPDH was used as an internal control.

We next determined whether GD55 induces more extensive apoptosis in PLC/PRF/5 sphere cells compared to ZD55. Hoechst 33342 staining assay disclosed that the fractions of nucleic fragmentations were raised more obviously in PLC/PRF/5 sphere cells treated with GD55 compared with ZD55 (Figure 5A). Additionally, expression level of anti-apoptosis protein XIAP and BCL-XL was decreased and the cleavage forms of PARP, caspase 3, caspase 8 and caspase 9 were enhanced in GD55-treated PLC/PRF/5 sphere cells, underlying that GD55 possessed stronger cytotoxic effect than ZD55 (Figure 5B).

### Antitumoral efficacy of GP73-mediated GD55 for liver sphere cells *in vivo*

**Figure 6:**
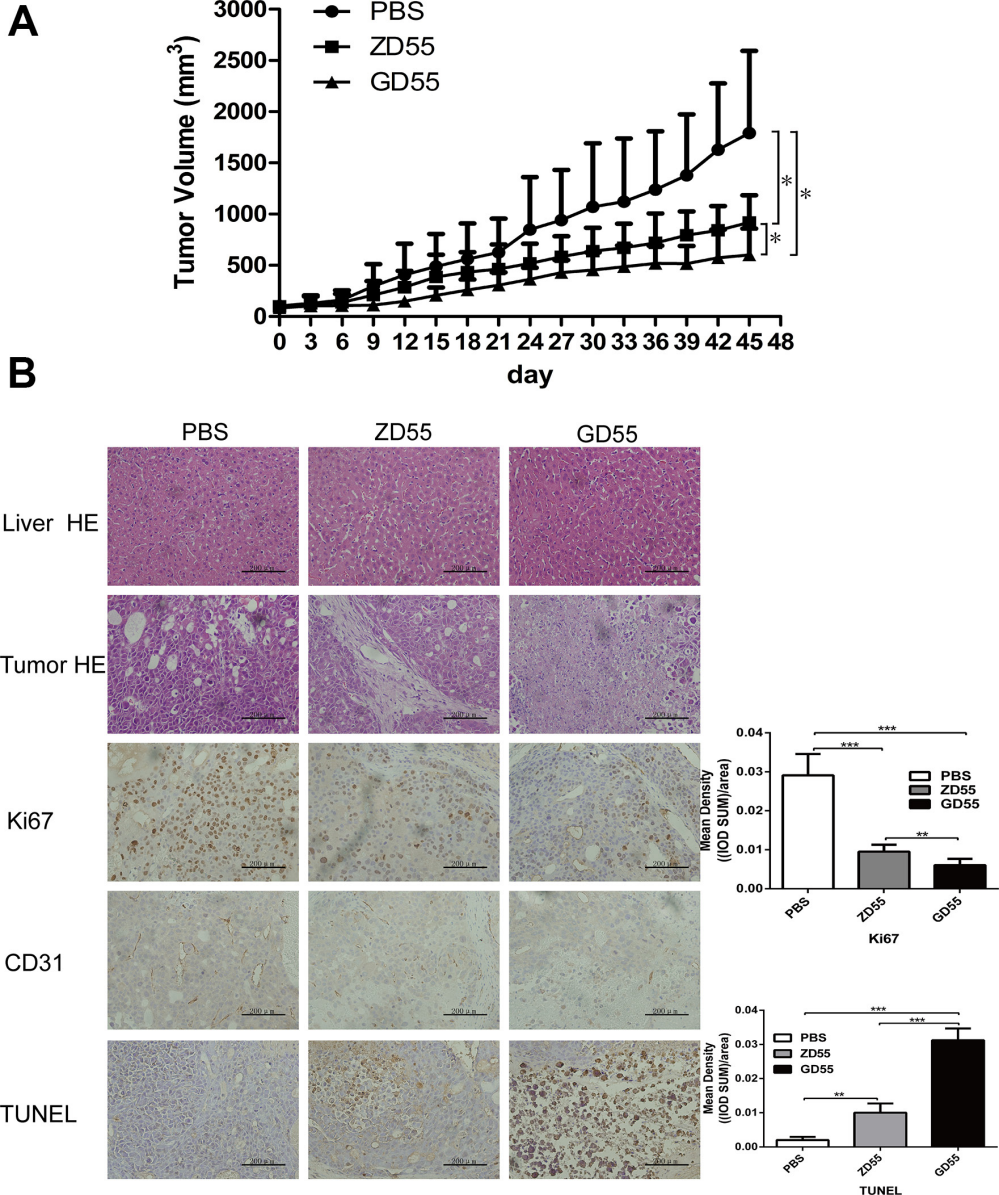
GD55 inhibited PLC/PRF/5 sphere xenograft tumors growth in nude mice (**A**) PLC/PRF/5 sphere cells were subcutaneously inoculated into female BALB/c nude mice at 2 × 10^6^ cells per mouse to tumor xenografts. When tumor volume reached 100 mm^3^, the mice were divided into three groups (*n* = 6) randomly and infected with 6 × 10^8^ pfu ZD55, GD55, or PBS every other day for repeated three times. The tumor volumes were monitored by periodic measurement. Tumor volume (V) was calculated according the formula: V (mm^3^) = length (mm) × width (mm)^2^/2. (**B**) Histological analysis of tumor sections in PBS, ZD55 and GD55 groups for PLC/PRF/5 sphere xenograft tumor. The upper two rows are hematoxylin and eosin staining analysis for animal liver and tumor tissues, indicating that hepatotoxicity or cell necrotic area in tumors. The middle row showed Ki67 and CD31 expression by IHC analysis in tumor tissues. The lower row is TUNEL assay for detecting apoptotic cells in tumor tissues treated by indicated treatment. GD55 induced more apoptosis of tumor cells. The brown color represents the apoptotic cells. Scale bar = 200 μm. A column drawing represent statistic data for mean density of 5 fields of view. Data was analyzed using IPP 6.0 image analysis software.

The data implicating the cytotoxic effect of GD55 on liver CSCs *in vitro* did not shed light on whether it plays a similar role *in vivo*, specifically on PLC/PRF/5 sphere cells. For this purpose, subcutaneous xenograft models on nude mice were established by inoculating PLC/PRF/5 sphere cells to test antitumoral efficacy of GD55. The results showed that xenograft tumours infected with GD55 presented the slowest growth rate compared to the PBS or ZD55 group (Figure 6A). Moreover, The histopathological test by hematoxylin and eosin (HE) staining showed that GD55 lead to more profound cell death and symptoms of necrosis in tumor mass than ZD55, and that GD55 didn't cause any obvious damage to liver tissue, which is essentially same to PBS-treated group (Figure 6B). Simultaneously, GD55 induced more obvious apoptosis of tumor cells detected by TUNEL assay, and strongly inhibited cell propogation and angiogenesis in xenograft tumors measured by IHC assay for Ki67 and CD31, respectively, relative to ZD55 (Figure 6B). The *in vivo* results suggested that GD55 could significantly inhibit the tumor growth derived from PLC/PRF/5 sphere cells, compared to common oncolytic virus ZD55.

## DISCUSSION

The present work reveals that GD55, a GP73-regulated oncolytic adenovirus, displays potent cytotoxic effect on liver CSC-like cells. ZD55, that had been extensively reported to possess significant cancer-killing effect, was further modified into The novel oncolytic adenovirus GD55was designed using GP73 promoter to promote the specificity and high efficiency of GD55 replication for hepatocellular carcinoma based on ZD55 [17], consistent with our results that GD55 exerted more excellent cytotoxic effect for liver CSCs-like cells than that of ZD55.

In addition to highly metastatic, drug-resistant and tumorigenic, CSCs are able to self-renew and divide asymmetrically to orchestrate the tumor mass, which are responsible for tumor recurrence [3]. With this concept, we examined liver CSCs-like cells derived from *in vitro*. Here, we used the suspension culture in serum-free medium supplemented with growth factors to enrich liver CSC-like cells (termed as sphere cells), as reported by others [23]. Our experiments showed that the sphere cells acquire the elevated mRNA and protein levels of the stemness genes, such as Nanog, Oct4, and Sox2 etc, which play central roles in the self-renewal and/or development of liver CSCs [24–26], and predict the prognosis of HCC patients [27, 28]. Sphere cells also upregulated the liver CSCs markers (such as CD24, CD133 and CD44 etc) and the markers (Twist, Slug, Fibronectin and N-cadherin etc) related to EMT that generates disseminating CSCs and contributes to the invasion-metastasis cascade of a tumor [29–31], or may acquire the enhanced chemoresistance reported recently [32], and yet down-regulated the mature hepatocyte markers (such as Albumin and G6P). Accordingly, the activated signaling pathways (such as AKT and STAT3) associated with liver CSCs were observed in the sphere cells compared with parental cells [26, 29]. In addition, the continuously expanding sphere cells still expressed the increased stemness genes, suggesting the capacity for self-renewal *in vitro*. Consistently, sphere cells were found to be more quiescent (higher percentage of G0/G1 phase cells)and to grow more slowly. Moreover, sphere cells were able to completely differentiate and revert back to their parental counterpart cells in serum medium adherently. Besides, we sufficiently verified that the sphere-forming cells possess the properties of liver CSC-like cells in many respects including the expression patterns of drug-resistence to convential drugs and sensitive to THO that specifically targets CSC and ZD55, high tumorigenicity and proliferative ability in NOD/SCID mice.

Despite aggressive treatment modalities, such as liver transplantation [33] and novel antimicrobial peptides [34], these strategies still failed to effectively eradicate CSCs within a tumor. The primary reason is that CSCs display the high expression levels of multi-drug resistant genes (as be confirmed by us) and the special capacity for expelling small molecule drugs [11]. Conversely, adenovirus can avoid this lack through its own infection mechanisms and do not be pumped out of CSCs, implying its potential application in tumor therapy [12, 13]. Actually, our results showed that the E1A proteins (the most important early gene for adenovirus replication) effectively expressed in GD55-infecting sphere cells in various MOIs and different time points, suggesting the replication of GD55 is not influenced by the resisting behavior of drug-resistence of liver CSC-like cells. Hence, GD55 performed a potent cytotoxicity effect on liver CSC-like cells. The killing effect of CSCs by GD55 was on average 50% higher than ZD55on PLC/PRF/5 sphere cells. *In vivo* experiments also confirmed that GP73-regulated GD55 held an increased inhibiting ability of tumor growth in BALB/c nude mice xenograft with liver sphere cells, to some extent through apoptosis inducement, anti-proliferative and anti-angiogenisis mechanisms within implanted tumors, which is consistent with some of the studies on other cancer-targeting oncolytic adenovirus types [35].

In addition, the capability of targeting liver CSCs of GD55 might be enhanced through further engineering modification. For example, it may be considered as a vessel to carry some liver CSC-specific inhibition genes or RNAi aiming to target the key transcription factors (such as Nanog) and the central signaling nodes (such as AKT/PI3K) based on the molecular differences between CSCs and non-CSCs. The introduction of immune promoting genes such as GM-CSF can promote the immune responses against tumors, and delivery of TRAIL or IL-24 gene effectively eliminates cancer cells through apoptosis, playing aim portant killing roles not only in CSCs, but also non-CSCs [8, 36]. Moreover, GP73, as a novel tissue biomarker, also is founded to occur in the development process of prostate cancer [37] and is down-regulated in gastric cancer and associated with tumor differentiation [38]; implying that GD55 has different effect for other types of cancers.

Recently, an interesting plasticity between non-CSC and CSC was observed in the breast carcinomas [39, 40], in which CSCs were able to give rise to non-CSCs progeny but also vice versa that the well-differentiated non-CSCs could readily convert to a CSC state. Indeed, the similar plasticity also was found to be occurring in normal epithelial tissues [41, 42]. It seems plausible that this plasticity perhaps robustly drives the metastatic dissemination of neoplastic cells and desease relapse, not only in breast cancer, but also maybe in other cancers. Thus, in consideration of this plasticity that might exactly exist in liver cancer [4], it is conceivable that the ideal agents (just like GD55) should have the following properties. They could effectively destroy non-CSCs, and more importantly, and they also simultaneously possess potent cytotoxicity on CSCs within a same tumor, which would completely eliminate the balance of the dynamics of interconversion between non-CSC and CSC, and ultimately eradicate tumor recurrence. These and other considerations suggest that GD55 might be considered as a promising therapeutic agent for liver cancer patients in clinic and, quite possibly, patients suffering other types of carcinomas.

## MATERIALS AND METHODS

### Cells line, sphere culture and sphere passage

PLC/PRF/5, Huh7, HepG2 and HEK293 cells were obtained from the cell Bank of Chinese Academy of Sciences (Shanghai, China) and maintained as a monolayer in DMEM (Gibco, USA) supplemented with 10% fetal bovine serum (Gibco, USA), 100 IU/ml of penicillin, and 100 μg/ml of streptomycin. All cells were incubated at 37°C in a humidified air atmosphere with 5% CO_2_ (Thermo, USA).

PLC/PRF/5, Huh7, HepG2 cells were collected and washed to remove serum, then suspended in free-serum DMEM/F12 (Gibco, USA) supplemented with 20 ng/ml human recombinant basic fibroblast growth factor (bFGF), 20 ng/ml human recombinant epidermal growth factor (EGF), 2% B27 supplement without vitamin A (Gibco, USA, an serum-free supplement), 100 IU/ml of penicillin and 100 μg/ml of streptomycin in Ultra Low Attachment 6-well plate (Coring). Fresh DMEM/F12 with growth factor was added to the Ultra Low Attachment plates every 2 days. Four days later, the tumors pheres were collected by gently centrifugation, then digested by Accutase (Sigma, USA) to single cell suspension for subsequent experiments.

Tumor spheres were digested to single cell, then centrifuged to remove the enzyme and resuspended with conditioned medium in Ultra Low Attachment 6-well plate allowed to reform spheres. The spheres were passaged every 4 days.

### Generation, purification and titration of adenovirus

The oncolytic adenovirus ZD55 and GD55 were previously constructed and stored in our laboratory. GD55 was constructed by using the GP73 promoter to replace the ZD55 E1A gene promoter. Viruses were amplified in HEK293 cells, followed by gradient CsCl solution centrifugation for purification. Moreover, virus titers were determined using the tissue culture infectious dose 50 assay in HEK293 cells.

### Colony formation

PLC/PRF/5 cells and PLC/PRF/5 sphere cells were digested to single cell, then seeded in DMEM with 10% FBS at a density of 3000 cells/well on 6-well plates. After 10 days, the cells were fixed in 4% paraformaldehyde and stained with crystal violet. The number of clones containing more than 70 cells was counted.

### Cytotoxicity assay

Cells were seeded at a density of 5000 cells/well in the 96-well plates and incubated for 12 h at 37°C prior to virus (ZD55, GD55) or drugs (Doxorubicin, Cisplatin, 5-Fuorouracil, Mitomycin) treatment. Then, MTT(3-(4, 5-dimethylthiazol-2-yl)-2, 5-diphenyltetrazolium bromide) solution in PBS (5 mg/ml, 20 μl) was added into each well. After the cells were incubated at 37°C for 4 h, the supernatant was removed and 150 μl dimethyl sylfoxide was added to each well. Cells viability was assessed by measuring the absorbance at 490 nm with a micro-plate reader after mixing thoroughly.

### Cytopathic assay

PLC/PRF/5 sphere cells were grown in a 48-well plate, then infected with ZD55 or GD55 at the indicated MOIs; uninfected cells served as control. Two days later, cells were exposed to 2% crystal violet in 20% methanol for 15 min and then washed with distilled water and documented by photography.

### Quantitative RT-PCR (qRT-PCR) assay

Total RNA was extracted by Trizol (Invitrogen, Carlsbad, CA, USA) according to the manufacturer's protocol and cDNA was synthesized with the ReverTra Ace qPCR RT Kit (Toyobo Osaka, Japan). Expression levels of genes were measured by SYBR Green Realtime PCR Master Mix (Toyobo) with their specific primers (Supplementary Table S1). GAPDH gene was used as an internal control.

### Western blot analysis

Total protein was extracted from treated cells using IP lysis buffer. Protein was then subjected to SDS-PAGE separation and electro-transferation to PVDF membranes. Primary antibodies against XIAP, Bcl-2, Survivin, DR5, STAT3, p-STAT3, β-Catenin, c-Myc, BCL-XL, AKT, p-AKT, caspase 8, caspase 9, PARP, GAPDH were purchased from Cell signaling technology. E1A and procaspase 3 were purchased from Santa Cruz. Albumin, OCT4, CD90, CD24, EpCAM, SOX2 were purchased from Antibody Revolution. GP73 was purchased from Abcam. All the secondary antibodies were purchased from Multi Sciences.

### Hoechst 33342 staining

PLC/PRF/5 sphere cells were seeded in 6-well culture plates and then infected with ZD55 or GD55 at the indicated MOIs; uninfected cells served as control. 48 h later, cells were stained with Hoechst 33342 (Beyotime, shanghai, China) at 1 mg/ml for 30 min at 37°C and observed under the inverted fluorescence microscope.

### Flow cytometric analysis

Cell cycle analysis was reflected by propidium iodide staining. Cells were collected and washed once with cold PBS. After fixation in 70% ethanol overnight at −20°C, cells were washed once with cold PBS and then stained with PI (Beyotime Biotech, China) at 37°C for 30 min. A fluorescence-activated cell sorting (FACS, Becton Dickioson) assay was immediately performed after staining.

### Animal experiments

All procedures with experimental animals were done according to the U.S. Public Health Service Policy on Human Care and the Use of Laboratory Animals. In tumorigenicity assay, Four-week-old male NOD/SCID mice were purchased from the Animal Research Committee of the Institute of Biochemistry and Cell Biology (Shanghai, China). The PLC/PRF/5 parental and sphere-forming cells were diluted at gradient concentrations and injected subcutaneously into the left and right flank of each mouse in serum-free DMEM/Matrigel (1:1) using 100 μl microsyringe. Total 5 × 10^4^ cells, 1 × 10^5^ cells, 2 × 10^5^ cells or 4 × 10^5^ cells were injected into each mouse and each group includes 2–3 mice.

To observe the anti-tumor effect of adenoviruses on PLC/PRF/5 sphere xenografts tumor, 2 × 10^6^ PLC/PRF/5 sphere cells in serum-free DMEM/Matrigel (2:1) were injected subcutaneously into right flank of each 5-week-old Female BALB/C nude mice (6 mice per group). When the xenograft tumors reached 100 mm^3^, mice were randomly divided into three groups (6 nude per group) and a total 6 × 10^8^ plaque-forming units (PFU) per mouse of ZD55 or GD55 or PBS were administrated via intratumoral injection once every other day for a total of three times. The tumour volume were measured every 3 days and calculated as length × width × width/2.

### HE staining, immunohistochemistry, and TUNEL assay

Excised tumor and liver samples were fixed by immersion in 4% paraformaldehyde, dehydrated with gradient ethanol, and embedded in paraffin. Tissue sections were stained with HE for morphological analysis. For immunohistochemical (IHC) analysis, tumor sections were incubated with monoclonal mouse anti-human Ki67 antibodies, monoclonal mouse anti-human GP73 antibodies and rabbit monoclonal anti-CD31 antibodies, respectively. The slides were then washed with PBS and incubated with avidin-biotin-peroxidase complex reagent (Vector Laboratories, Burlingame, CA) and detected with diamino- benzidinetetra- hydrochloride solution. Apoptotic cells in tumor tissue sections were assessed by TUNEL staining with a TACS TdT kit *in situ* apoptosis detection kit (Sino-American Biotechnology Co., Luoyang, China) according to the manufacturer's protocol. All sections were counterstained with hematoxylin and examined with bright-field microscopy. Immunohistochemical staining and TUNEL assay were quantitated using IPP 6.0 image analysis software (Media Cybernetics, USA), and 5–8 fields of view were selected on each section and photographed. Mean density were calculated using the following formula: Mean Density = (IOD SUM)/area.

### Statistical analysis

The data are represented by the mean ± SD. Differences among the different treatment groups were assessed by analysis of variance and student's *t* test. The difference between data were considered to be statistically significant when *P* < 0.05 (*), to be very significant when *p* < 0.01 (**) and to be very much significant when *p* < 0.001 (***).

### SUPPLEMENTARY MATERIALS FIGURES AND TABLE



## References

[1] Magee JA, Piskounova E, Morrison SJ (2012). Cancer stem cells: impact, heterogeneity, and uncertainty. Cancer Cell.

[2] Visvader JE, Lindeman GJ (2012). Cancer stem cells: current status and evolving complexities. Cell stem cell.

[3] Meacham CE, Morrison SJ (2013). Tumour heterogeneity and cancer cell plasticity. Nature.

[4] Yamashita T, Wang XW (2013). Cancer stem cells in the development of liver cancer. J Clin Invest.

[5] Jemal A, Bray F, Center MM, Ferlay J, Ward E, Forman D (2011). Global cancer statistics. CA Cancer J Clin.

[6] Zhang F, Chen XP, Zhang W, Dong HH, Xiang S, Zhang WG, Zhang BX (2008). Combined hepatocellular cholangiocarcinoma originating from hepatic progenitor cells: immunohistochemical and double-fluorescence immunostaining evidence. Histopathology.

[7] Pattabiraman DR, Weinberg RA (2014). Tackling the cancer stem cells - what challenges do they pose?. Nat Rev Drug Discov.

[8] Russell SJ, Peng KW, Bell JC (2012). Oncolytic virotherapy. Nat Biotechnol.

[9] Guo ZS, Bartlett DL (2014). Oncolytic viruses as platform for multimodal cancer therapeutics: a promising land. Cancer Gene Ther.

[10] Cripe TP, Wang PY, Marcato P, Mahller YY, Lee PW (2009). Targeting cancer-initiating cells with oncolytic viruses. Mol Ther.

[11] Dean M, Fojo T, Bates S (2005). Tumour stem cells and drug resistance. Nat Rev Cancer.

[12] Yang Y, Xu H, Shen J, Yang Y, Wu S, Xiao J, Xu Y, Liu XY, Chu L (2015). RGD-modifided oncolytic adenovirus exhibited potent cytotoxic effect on CAR-negative bladder cancer-initiating cells. Cell Death & Dis.

[13] Yang Y, Xu H, Huang W, Ding M, Xiao J, Yang D, Li H, Liu XY, Chu L (2015). Targeting lung cancer stem-like cells with TRAIL gene armed oncolytic adenovirus. J Cell Mol Med.

[14] Liu XY, Gu JF, Shi WF (2005). Targeting gene-virotherapy for cancer. Acta Biochim Biophys Sin.

[15] Liu XY, Gu JF (2006). Targeting gene-virotherapy of cancer. Cell research.

[16] Zhang ZL, Zou WG, Luo CX, Li BH, Wang JH, Sun LY, Qian QJ, Liu XY (2003). An armed oncolytic adenovirus system, ZD55-gene, demonstrating potent antitumoral efficacy. Cell Res.

[17] Wang Y, Liu T, Huang P, Zhao H, Zhang R, Ma B, Chen K, Huang F, Zhou X, Cui C, Liu X (2015). A novel Golgi protein (GOLPH2)-regulated oncolytic adenovirus exhibits potent antitumor efficacy in hepatocellular carcinoma. Oncotarget.

[18] Zhou Y, Yin X, Ying J, Zhang B (2012). Golgi protein 73 versus alpha-fetoprotein as a biomarker for hepatocellular carcinoma: a diagnostic meta-analysis. BMC Cancer.

[19] Collura A, Marisa L, Trojan D, Buhard O, Lagrange A, Saget A, Bombled M, Mechighel P, Ayadi M, Muleris M, de Reynies A, Svrcek M, Flejou JF (2013). Extensive characterization of sphere models established from colorectal cancer cell lines. Cell Mol Life Sci.

[20] Singh SK, Clarke ID, Terasaki M, Bonn VE, Hawkins C, Squire J, Dirks PB (2003). Identification of a cancer stem cell in human brain tumors. Cancer Res.

[21] Dontu G, Abdallah WM, Foley JM, Jackson KW, Clarke MF, Kawamura MJ, Wicha MS (2003). *In vitro* propagation and transcriptional profiling of human mammary stem/progenitor cells. Gene Dev.

[22] Sachlos E, Risueno RM, Laronde S, Shapovalova Z, Lee JH, Russell J, Malig M, McNicol JD, Fiebig-Comyn A, Graham M, Levadoux-Martin M, Lee JB, Giacomelli AO (2012). Identification of drugs including a dopamine receptor antagonist that selectively target cancer stem cells. Cell.

[23] Cao L, Zhou Y, Zhai B, Liao J, Xu W, Zhang R, Li J, Zhang Y, Chen L, Qian H, Wu M, Yin Z (2011). Sphere-forming cell subpopulations with cancer stem cell properties in human hepatoma cell lines. BMC Gastroenterol.

[24] Shan J, Shen J, Liu L, Xia F, Xu C, Duan G, Xu Y, Ma Q, Yang Z, Zhang Q, Ma L, Liu J, Xu S (2012). Nanog regulates self-renewal of cancer stem cells through the insulin-like growth factor pathway in human hepatocellular carcinoma. Hepatology.

[25] Lin CY, Loven J, Rahl PB, Paranal RM, Burge CB, Bradner JE, Lee TI, Young RA (2012). Transcriptional amplification in tumor cells with elevated c-Myc. Cell.

[26] Lee TK, Castilho A, Cheung VC, Tang KH, Ma S, Ng IO (2011). CD24(+) liver tumor-initiating cells drive self-renewal and tumor initiation through STAT3-mediated NANOG regulation. Cell Stem Cell.

[27] Yin X, Li YW, Zhang BH, Ren ZG, Qiu SJ, Yi Y, Fan J (2012). Coexpression of stemness factors Oct4 and Nanog predict liver resection. Annals of surgical oncology.

[28] Huang P, Qiu J, Li B, Hong J, Lu C, Wang L, Wang J, Hu Y, Jia W, Yuan Y (2011). Role of Sox2 and Oct4 in predicting survival of hepatocellular carcinoma patients after hepatectomy. Clinical biochemistry.

[29] Ji J, Wang XW (2012). Clinical implications of cancer stem cell biology in hepatocellular carcinoma. Semi Oncol.

[30] Liu AY, Cai Y, Mao Y, Lin Y, Zheng H, Wu T, Huang Y, Fang X, Lin S, Feng Q, Huang Z, Yang T, Luo Q (2014). Twist2 promotes self-renewal of liver cancer stem-like cells by regulating CD24. Carcinogenesis.

[31] Mani SA, Guo W, Liao MJ, Eaton EN, Ayyanan A, Zhou AY, Brooks M, Reinhard F, Zhang CC, Shipitsin M, Campbell LL, Polyak K, Brisken C (2008). The epithelial-mesenchymal transition generates cells with properties of stem cells. Cell.

[32] Fischer KR, Durrans A, Lee S, Sheng J, Li F, Wong ST, Choi H, El Rayes T, Ryu S, Troeger J, Schwabe RF, Vahdat LT, Altorki NK (2015). Epithelial-to-mesenchymal transition is not required for lung metastasis but contributes to chemoresistance. Nature.

[33] Chen K, Man K, Metselaar HJ, Janssen HL, Peppelenbosch MP, Pan Q (2014). Rationale of personalized immunosuppressive medication for hepatocellular carcinoma patients after liver transplantation. Liver Transpl.

[34] Lu Y, Zhang TF, Shi Y, Zhou HW, Chen Q, Wei BY, Wang X, Yang TX, Chinn YE, Kang J, Fu CY (2016). PFR peptide, one of the antimicrobial peptides identified from the derivatives of lactoferrin, induces necrosis in leukemia cells. Sci Rep.

[35] Thaci B, Ulasov IV, Ahmed AU, Ferguson SD, Han Y, Lesniak MS (2013). Anti-angiogenic therapy increases intratumoral adenovirus distribution by inducing collagen degradation. Gene Ther.

[36] Liu XY (2012). The Excellent Anti-Tumour Strategy (CTGVT, OV-gene) and the Excellent Anti-Tumor Gene (IL-24). International journal of biomedical science.

[37] Kristiansen G, Fritzsche FR, Wassermann K, Jager C, Tolls A, Lein M, Stephan C, Jung K, Pilarsky C, Dietel M, Moch H (2008). GOLPH2 protein expression as a novel tissue biomarker for prostate cancer: implications for tissue-based diagnostics. Brit J Cancer.

[38] Chen LG, Wang HJ, Yao HB, Guan TP, Wu F, He XJ, Ma YY, Tao HQ, Ye ZY (2013). GP73 is down-regulated in gastric cancer and associated with tumor differentiation. World J Surg Oncol.

[39] Chaffer CL, Marjanovic ND, Lee T, Bell G, Kleer CG, Reinhardt F, D'Alessio AC, Young RA, Weinberg RA (2013). Poised chromatin at the ZEB1 promoter enables breast cancer cell plasticity and enhances tumorigenicity. Cell.

[40] Chaffer CL, Brueckmann I, Scheel C, Kaestli AJ, Wiggins PA, Rodrigues LO, Brooks M, Reinhardt F, Su Y, Polyak K, Arendt LM, Kuperwasser C, Bierie B (2011). Normal and neoplastic nonstem cells can spontaneously convert to a stem-like state. Proc Natl Acad Sci U S A.

[41] Tata PR, Mou H, Pardo-Saganta A, Zhao R, Prabhu M, Law BM, Vinarsky V, Cho JL, Breton S, Sahay A, Medoff BD, Rajagopal J (2013). Dedifferentiation of committed epithelial cells into stem cells *in vivo*. Nature.

[42] Stange DE, Koo BK, Huch M, Sibbel G, Basak O, Lyubimova A, Kujala P, Bartfeld S, Koster J, Geahlen JH, Peters PJ, van Es JH, van de Wetering M (2013). Differentiated Troy + chief cells act as reserve stem cells to generate all lineages of the stomach epithelium. Cell.

