# Supplementary material for: GP73-regulated oncolytic adenoviruses possess potent killing effect on human liver cancer stem-like cells
Source: Oncotarget. 2016 Apr 18;7(20):29346–58. doi: 10.18632/oncotarget.8830 (PMC5045400; doi:10.18632/oncotarget.8830)
Supplement: Supplementary file 1 [file oncotarget-07-29346-s001.pdf]

GP73-regulated oncolytic adenoviruses possess potent killing effect on human liver cancer stem-like cells

Supplementary Materials

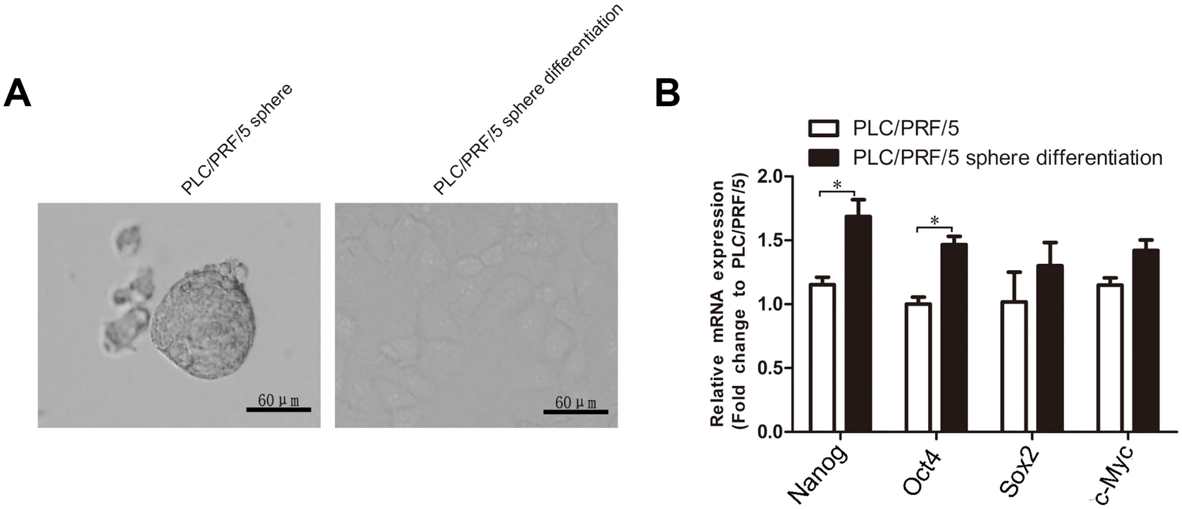

Supplementary Figure S1: The attributes of differentiation for PLC/PRF/5 sphere cells. (**A**) PLC/PRF/5 sphere cells differentiated to their parental counterparts through culturing adherently in 10% FBS medium by 7 days. scale bar: 200 μm.(**B**) PLC/PRF/5 sphere cells differentiated by 4 days in 5% FBS medium adherently still elevated liver CSCs-assaciated genes. All experiments were repeated three times and all data shown represented mean ± SD ($n = 3$). *$P < 0.05$, **$P < 0.01$, ***$P < 0.001$.

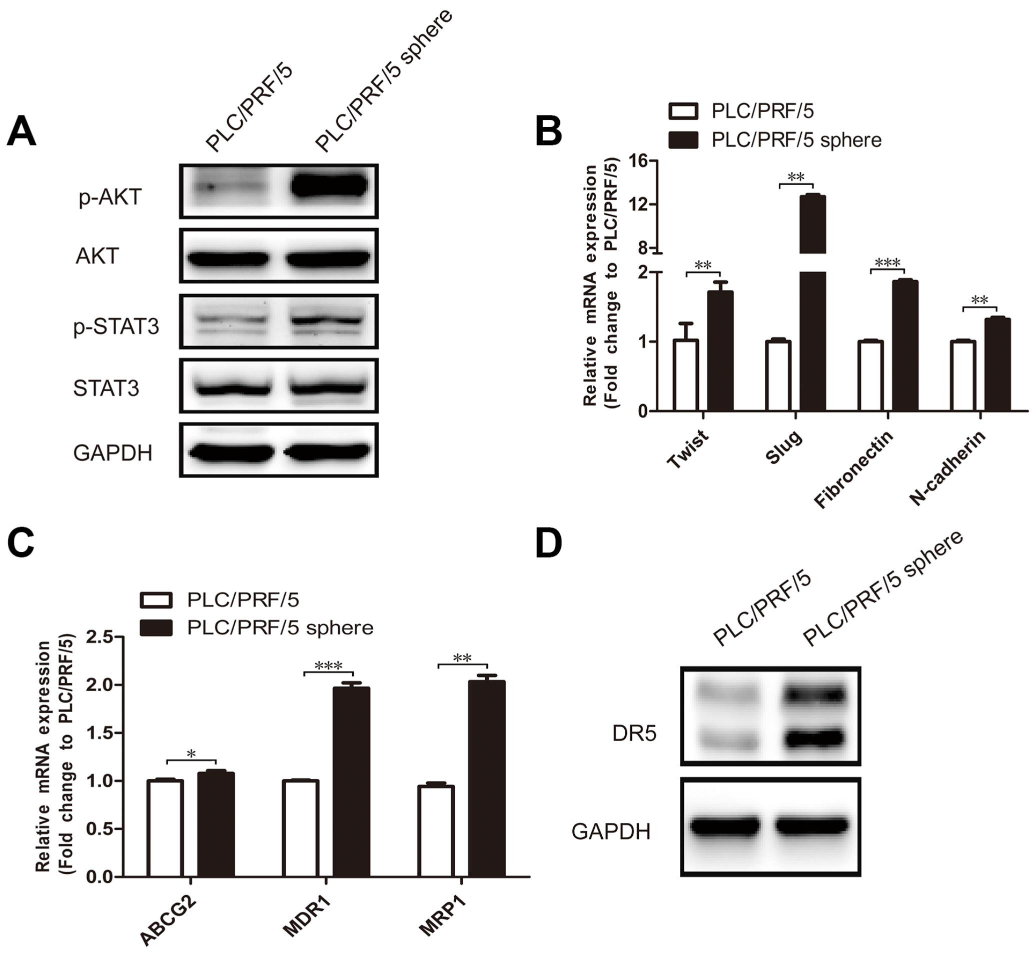

Supplementary Figure S2: PLC/PRF/5 sphere cells upregulated the related genes and proteins assosiated with CSCs. (**A**) Western blot indicated that sphere cells activated PI3K/AKT signaling pathway and increased phosphorylation levels of STAT3 than parental cells in PLC/PRF/5. (**B**) PLC/PRF/5 sphere cells overexpressed the epithelial-mesenchymal transition-inducing markers (e.g., Fibronectin and N-Cadherin) and transcription factors (e.g., Twist and Slug) when compared to PLC/PRF/5 cells. qRT-PCR data were normalized to GAPDH gene and shown as fold change relative to PLC/PRF/5 cells. (**C**) Upregulation of the related mRNAs of drug-resistance (e.g., ABCG2, MDR1, MRP1) in PLC/PRF/5 sphere cells. qRT-PCR data were normalized to GAPDH gene and are shown as fold change relative to PLC/PRF/5 cells. (**D**) DR5 (dopamine receptors 5) protein level increased in PLC/PRF/5 sphere cells. All experiments were repeated three times and all data shown represented mean ± SD (n = 3). *$P < 0.05$, **$P < 0.01$, ***$P < 0.001$.

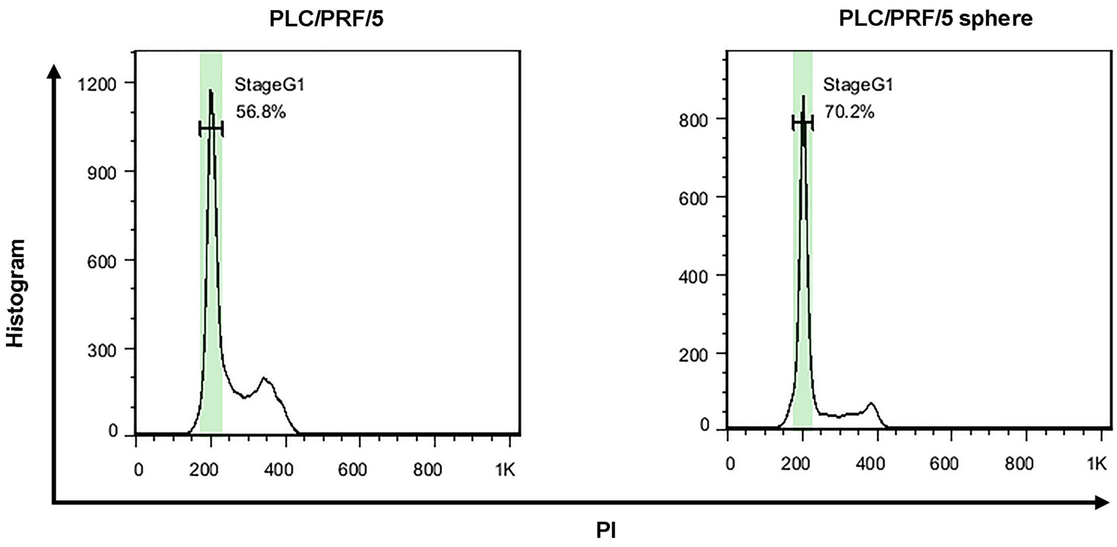

Supplementary Figure S3: Cell cycle distribution of PLC/PRF/5 cells and PLC/PRF/5 sphere cells. Numbers indicate the proportion of G0/G1 phase cells.

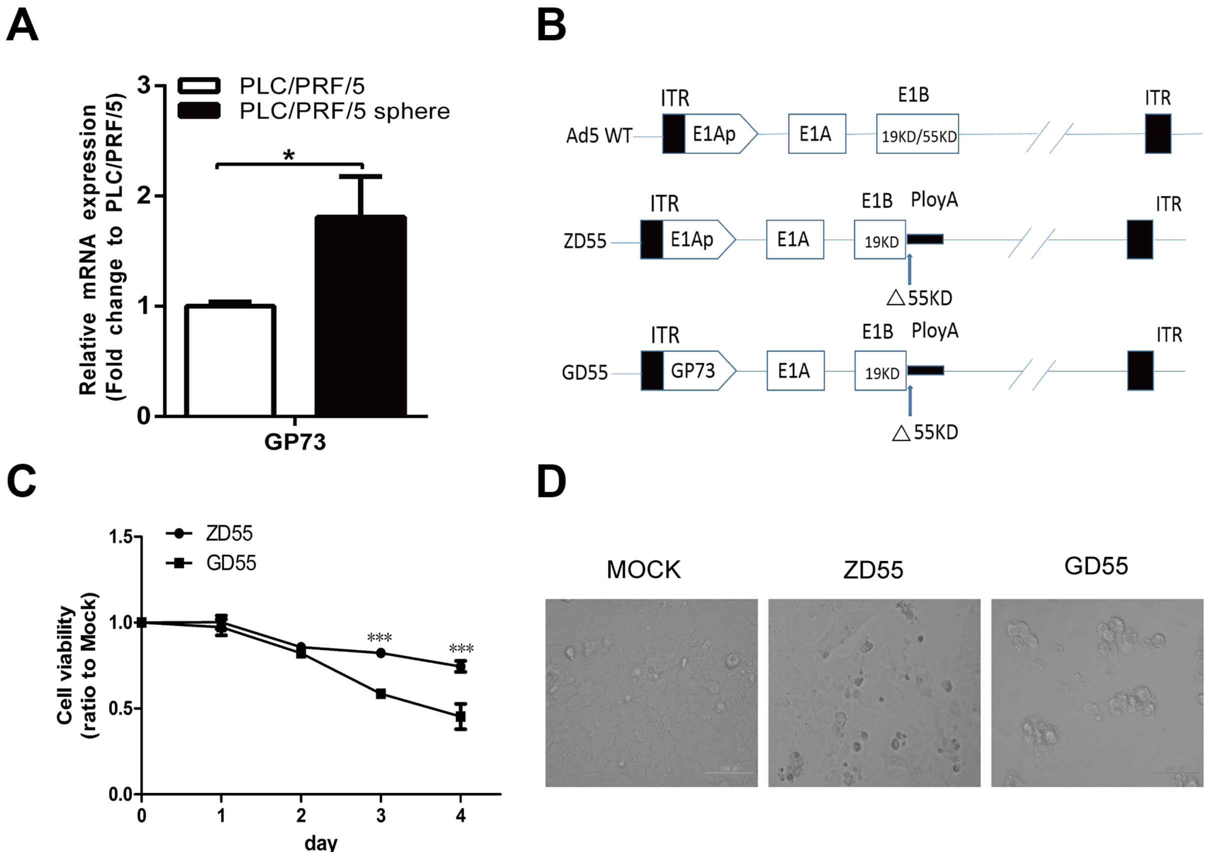

Supplementary Figure S4: Construction of adenoviruses and cytotoxic effect of GD55 on PLC/PRF/5 sphere cells. (**A**) PLC/PRF/5 sphere cells acquired a little more expression level of GP73 when compared to PLC/PRF/5 cells. qRT-PCR data were normalized to GAPDH gene and shown as fold change relative to PLC/PRF/5 cells. (**B**) Schematic structure of recombinant oncolytic adenovirus was compared to the wild type adenovirus (Ad5WT). GD55 was designed with E1A promoter replaced by *GOLPH2* core promoter and E1B 55 kDa gene deletion. (**C**) Morphological observation of PLC/PRF/5 sphere cells after treated with ZD55 and GD55 (5MOI) for 72 h. scale bar: 200 μm. (**D**) PLC/PRF/5 sphere ells were infected with GD55 or ZD55 at a MOI of 5. On the day 1, 2, 3, and 4 postinfection, cell viability was measured with MTT assay. All experiments were repeated three times and all data shown represented mean ± SD (n = 3). *P < 0.05, **P < 0.01, ***P < 0.001.

Supplementary Table S1: Sequences of Primers

Primers	Sequences
Nanog-forward	5′- CTCTCCTCTTCCTTCCTCCAT-3′
Nanog-reverse	5′- TTGCGACACTCTTCTCTGC-3′
Oct4-forward	5′- CTTGCTGCAGAAGTGGGTGGAGGAA -3′
Oct4-reverse	5′- CTGCAGTGTGGGTTTCGGGCA-3′
Sox2-forward	5′- AAATGGGAGGGGTGCAAAAGAGGAG -3′
Sox2-reverse	5′- CAG CTG TCA TTT GCT GTG GGT GAT G-3′
CD44-forward	5′- CGGACACCATGGACAAGTTT-3′
CD44-reverse	5′- GAAAGCCTTGCAGAGGTCAG-3′
CD90-forward	5′- GACCCGTGAGACAAAGAAGC-3′
CD90-reverse	5′- GCCCTCACACTTGACCAGTT-3′
Albumin-forward	5′- CACAAAGATGACAACCCAAACCTCC-3′
Albumin-reverse	5′- GGAGTTCCGGGGCATAAAAGTAAG-3′
GAPDH-forward	5′-GATTTGGTCGTATTGGGCG-3′
GAPDH-reverse	5′-TGGAAGATGGTGATGGGAT-3′
c-Myc-forward	5′- GCACTGGAACTTACAACAC-3′
c-Myc-reverse	5′- GGAGAGCCTTTCAGAGAAG-3′
CD133-forward	5′-AAGCATTGGCATCTTCTATGG-3′
CD133-reverse	5′-AAGCACAGAGGGTCATTGAGA-3′
EpCAM-forward	5′-GCGGCTCAGAGAGACTGTG -3′
EpCAM-reverse	5′-CCAAGCATTTAGACGCCAGTTT-3′
CD24-forward	5′-TGAAGAACATGTGAGAGGTTTGAC-3′
CD24-reverse	5′-GAAAACTGAATCTCCATTCCACAA-3′
ABCG2-forward	5′- GTTCTCAGCAGCTCTTCGGCTT -3′
ABCG2-reverse	5′- TCCTCCAGACACACCACGGATA -3′
MDR1-forward	5′- CATTGGTGTGGTGAGTCAG -3′
MDR1-reverse	5′- GTCATAGGCATTGGCTTCC -3′
MRP1-forward	5′-AAGACCAAGACGTATCAGGT-3′
MRP1-reverse	5′-CAATGGTCACGTAGACGGCAA-3′
Fibronectin-forward	5′- CAGTGGGAGACCTCGAGAAG -3′
Fibronectin-reverse	5′- TCCCTCGGAACATCAGAAAC -3′

N-Cadherin-forward	5′- ACAGTGGCCACCTACAAAGG-3′
N-Cadherin -reverse	5′- CCGAGATGGGGTTGATAATG-3′
G6P-forward	5′-GTCTGTCACGAATCTACCTTG-3′
G6P-reverse	5′-CTACACCCAGTCCCTTGAG-3′
Twist-forward	5′-AAGGCATCACTATGGACTTTCTCT-3′
Twist-reverse	5′-GCCAGTTTGATCCCAGTATTTT-3′
Slug-forward	5′-GCATTTCTTCACTCCGAAGC-3′
Slug-reverse	5′-TGAATTCCATGCTCTTGCAG-3′
GP73-forward	5′-GTGTGAGGAGCGAATAGAAGAGG-3′
GP73-reverse	5′-GTCTCTGGTCGTTGTTTTCACT-3′
